# Shared somatosensory and motor functions in musicians

**DOI:** 10.1038/srep37632

**Published:** 2016-11-25

**Authors:** Moe Hosoda, Shinichi Furuya

**Affiliations:** 1 Musical Skill and Injury Center (MuSIC), Sophia University, Tokyo, JAPAN

## Abstract

Skilled individuals are characterized by fine-tuned perceptual and motor functions. Here, we tested the idea that the sensory and motor functions of highly-trained individuals are coupled. We assessed the relationships among multifaceted somatosensory and motor functions of expert pianists. The results demonstrated a positive covariation between the acuity of weight discrimination and the precision of force control during piano keystrokes among the pianists but not among the non-musicians. However, neither the age of starting musical training nor the total amount of life-long piano practice was correlated with these sensory-motor functions in the pianists. Furthermore, a difference between the pianists and non-musicians was absent for the weight discrimination acuity but present for precise force control during keystrokes. The results suggest that individuals with innately superior sensory function had finer motor control only in a case of having undergone musical training. Intriguingly, the tactile spatial acuity of the fingertip was superior in the pianists compared with the non-musicians but was not correlated with any functions representing fine motor control among the pianists. The findings implicate the presence of two distinct mechanisms of sensorimotor learning elicited by musical training, which occur either independently in individual sensorimotor modalities or through interacting between modalities.

Plasticity of the nervous system changes sensory and motor functions through training. Musicians provide unique opportunities to shed light on the complex biological mechanism underlying the interaction of neuroplasticity of the human sensorimotor system with long-term multimodal training from childhood^1^,^2^,^3^. Previous studies evidenced use-dependent plastic changes in both the structure and function of the somatosensory cortex in musicians^4^,^5^. A two-point discrimination test further identified superior tactile perception of the fingertips in musicians compared with non-musicians^6^,^7^. A similar finding was also reported in the motor domain, in which finer motor control of musicians relative to non-musicians is associated with neuroplastic changes in motor-related regions^8^,^9^. A longitudinal study also demonstrated an improvement in fine motor control through extensive piano practice, thus indicating a causal effect of musical training on motor dexterity^10^. However, what remains unknown is to what extent the somatosensory functions of trained individuals play a role in fine motor control, and vice versa.

Recent research has revealed a shared mechanism between motor and perceptual learning^11^,^12^,^13^. For example, in speech production, adaptation to altered auditory feedback and altered somatosensory feedback results in perceptual shifts^14^,^15^. In contrast, perceptual training, such as passive limb movement, directly enhances motor learning^16^,^17^,^18^. In addition, exposure to extensive tactile stimulation enhances not only tactile acuity but also manual motor dexterity^19^,^20^. These empirical findings support the notion that perceptual and motor learning generally occur together. A reciprocal link between neuroplasticity in somatosensory and motor systems corroborates the concept of error-feedback learning^21^, which denotes that sensory feedback signaling motor errors facilitate the accuracy of goal-directed movements with training. Thus, error-feedback learning envisages that higher sensory acuity provides finer-tuned sensory feedback through trial-and-error learning, which leads to superior movement precision. However, recent studies also demonstrated key roles of genetic predisposition in sensorimotor skill^22^,^23^,^24^,^25^,^26^. This predicts that even if sensory and motor functions are coupled among trained individuals, either or both of the functions do not necessarily result from training^27^.

The purpose of the study was to assess the relationships among the somatosensory and motor functions of musicians. A spectrum of these functions were evaluated and compared within and between expert musicians and non-musicians. We postulated superior somatosensory and motor functions in musicians compared with non-musicians and a correlation between somatosensory acuity and fine motor control in musicians. The study also sought to assess the effects of early and deliberate musical practice on somatosensory and motor functions.

## Results

Forty participants including twenty-one pianists and nineteen non-musicians underwent five independent sensorimotor function tests (passive and active sensory function tests, and music-relevant and music-irrelevant motor function tests). Each of the tests was performed with the participant’s non-dominant ring finger, and assessed a variety of sensorimotor functions.

**Figure 1:**
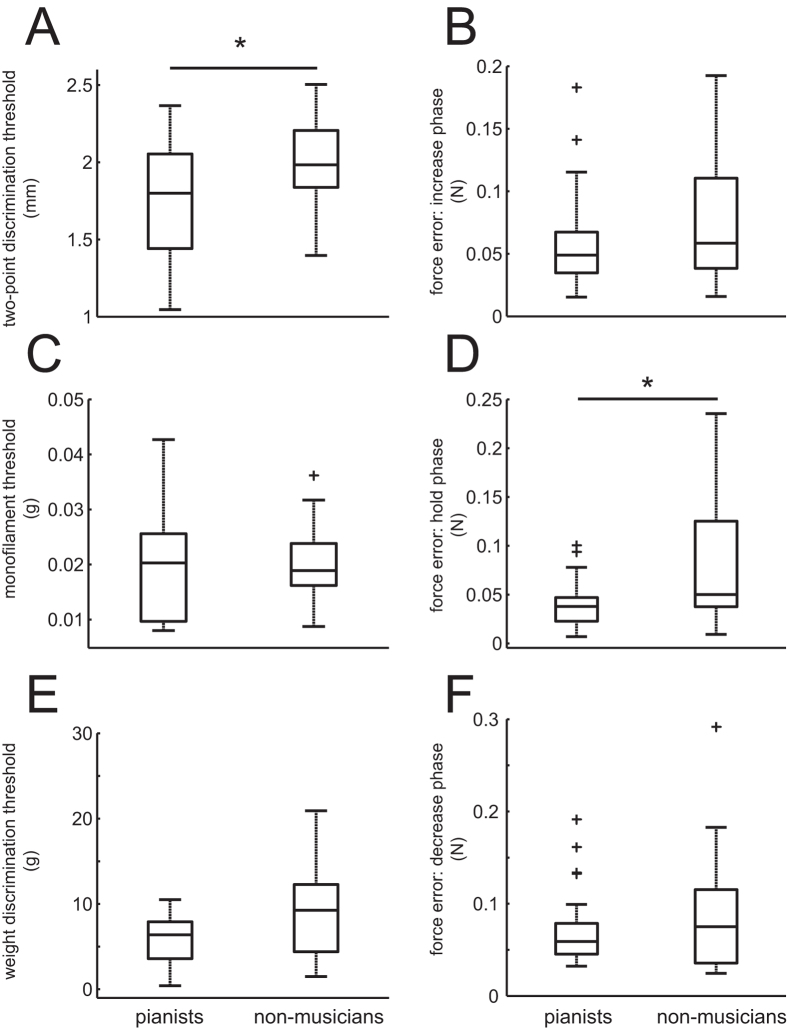
Group means of the music-irrelevant somatosensory and motor functions of the tip of the left ring finger in the pianists (left plots) and the non-musicians (right plots). Left panel: somatosensory acuity of the left ring fingertip. (**A**) Tactile spatial acuity threshold measured by the two-point discrimination test, (**C**) passive force discrimination threshold measured by monofilaments, (**E**) weight discrimination threshold measured by successively comparing the weights of two objects. Right panel: Error of the isometric force production with the left ring finger during tracking a visually displayed trajectory at the phases of (**B**) increasing, (**D**) holding, and (**F**) decreasing force.

Figure 1 displays the group means for each of the music-irrelevant sensory (left panel) and motor (right panel) tests in the musicians and the non-musicians. Of the sensory tests, only the two-point discrimination test exhibited a significantly lower threshold value in the pianists compared to in the non-musicians (p = 0.027). Neither the passive force discrimination nor weight discrimination tests yielded a significant group difference (p = 0.722 and p = 0.076, respectively). Concerning the motor tests, the error between the target and exerted forces was significantly smaller for the pianists than for the non-musicians only during the hold phase (p = 0.013). The error was not smaller during the force increasing and decreasing phases (p = 0.273 and p = 0.394, respectively). The results confirmed that the musicians are superior to the non-musicians in some but not all of the somatosensory and motor functions that are irrelevant to musical performance.

**Figure 2:**
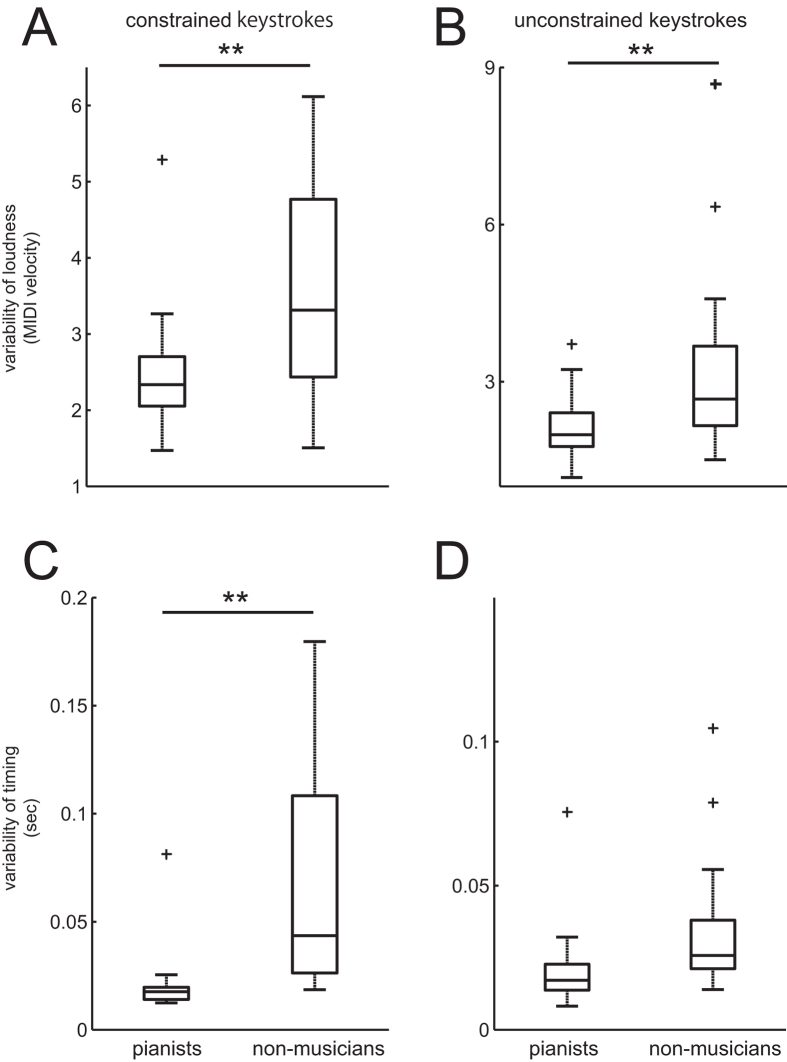
Group means of the music-relevant motor functions during the constrained (left panel) and unconstrained (right panel) piano keystrokes with the left ring finger in the pianists (left plots) and the non-musicians (right plots). The variability of loudness (top panel) and timing (bottom panel).

Figure 2 illustrates the group means for each of the music-relevant motor tests through the repetitive piano keystrokes in the constrained (left panel) and unconstrained (right panel) conditions in the musicians and the non-musicians. The inter-strike variability of loudness was significantly smaller for the pianists than for the non-musicians in both the constrained (p = 0.003) and unconstrained (p = 0.010) conditions. The inter-strike variability of the timing of the keystrokes was also significantly less for the pianists than the non-musicians for the constrained condition (p = 0.002) but not the unconstrained condition (p = 0.078). Overall, the results confirmed more precise control of timing and force during the music-relevant tests for the musicians compared with the non-musicians.

**Figure 3:**
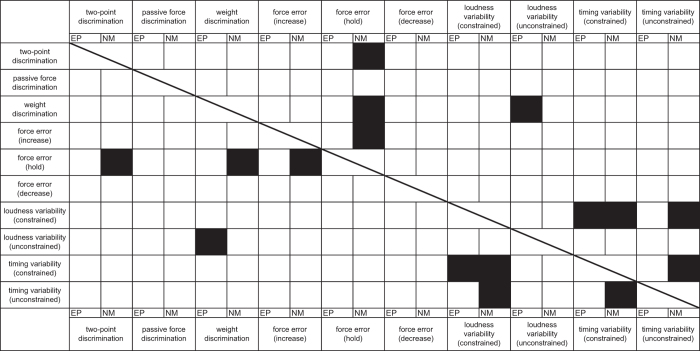
A matrix representing a significant covariation between two pairs of the sensory and motor functions for each of the expert pianists (EP) and non-musicians (NM). A black cell indicates a significant partial regression coefficient derived from the multiple regression analysis (p < 0.05) (i.e. Tables 1 and 2).

**Figure 4:**
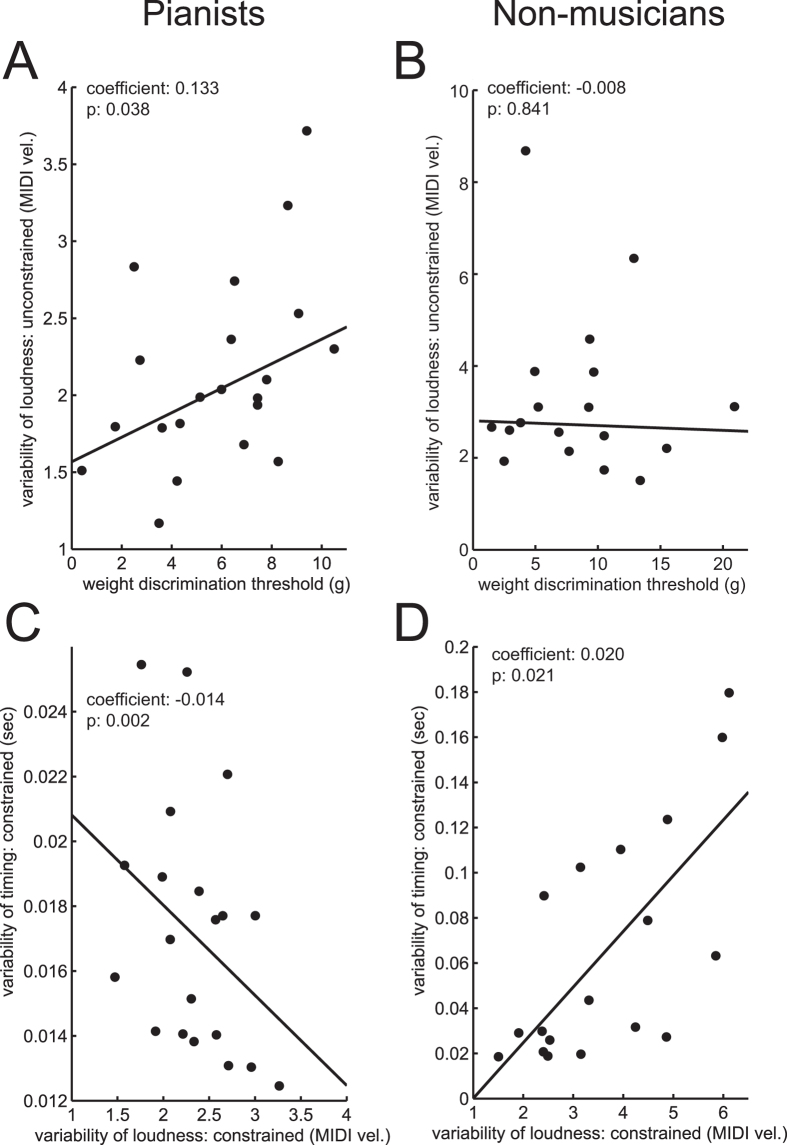
Scatter plots between some representative somatosensory and motor functions among the pianists (left panel) and the non-musicians (right panel). (**A,B**) The weight discrimination threshold versus the variability of loudness of unconstrained piano keystrokes, and (**C,D**) the variability of timing and loudness during the constrained piano keystrokes.

To assess the relationships among the somatosensory and motor abilities in the music-relevant and music-irrelevant tests, a multiple linear regression analysis was performed for each of the variables evaluated among the individuals of the musician and non-musician groups. The analysis was performed separately for each of the two groups owing both to the aforementioned group difference in some of the variables and to no hypothesis of differences in the regression coefficient between the groups. Tables 1 and 2 summarize the results. To highlight a group difference in significant predictors of each of the sensory and motor functions, a correlation matrix was also depicted based on Tables 1 and 2 (Fig. 3). Some representative examples are plotted in Fig. 4. Among the pianists, there was a significant positive regression between the weight discrimination threshold and inter-strike variability of loudness in the unconstrained keystroke tasks (Fig. 4, top-left). This result indicates a relationship between the music-irrelevant somatosensory and music-related motor functions. By contrast, this relationship was not evident in the non-musicians (Fig. 4, top-right). There was also a significant regression between the inter-strike variability of timing and loudness, which was negative and positive in terms of the relationship for the pianists (Fig. 4, bottom-left) and the non-musicians (Fig. 4, bottom-right), respectively.

The questionnaire that evaluated the participants’ history of musical training was only administered to the group of pianists. The results indicated that the average age at which musical training commenced was 4.1 ± 0.9 (range: 3–6) years old and that the total amount of piano practice during a lifetime was 21915 ± 11561 (range: 6570–53290) hours. A multiple linear regression analysis predicting each of the somatosensory and motor functions assessed by the individual tests yielded no significant covariation with each of the two variables representing the history of musical training (p > 0.05). Therefore, these results indicate that the individual differences in musical training history among the pianists failed to account for the inter-individual variability of the sensory and motor functions evaluated in the study.

## Discussion

The present study demonstrated superior abilities in several particular somatosensory and motor functions in trained pianists compared with musically untrained individuals. In agreement with several previous findings^6^,^7^, the two-point discrimination threshold of the trained pianists was lower than that of the non-musicians, confirming enhanced spatial tactile acuity (Fig. 1A) in the trained pianists. A novel observation of the study was the lack of effects of musical expertise on both the cutaneous pressure threshold and the weight sensibility threshold (Fig. 1C,E), which indicates no enhancement in the active and passive force discrimination abilities through extensive piano practice. The findings suggest that piano training drives plastic adaptation of the somatosensory system that is specifically responsible for spatial tactile acuity. This finding is not obvious because playing the piano involves repetition of pressing and releasing piano keys with various forces, which may well assume enhancement of force discrimination abilities rather than spatial tactile acuity through piano training. In the motor domain, the pianists exhibited a lower error of both sustained force production in a music-irrelevant task and dynamic force production in a music-relevant task compared with the non-musicians. The functional dissociation between the perception and production of force may imply that the superior controllability of force in the pianists compared with the non-musicians is not tightly associated with tactile spatial acuity. However, in most cases of depressing a piano key, pianists slide their fingertip on a key surface along the anterior-posterior axis^28^. Therefore, the spatial tactile perception detects an incremental spatial change of the fingertip location during the key depression, which suggests a role of superior spatial tactile acuity in fine motor control in pianists. Presumably, musical training modulates the somatosensory functions in an instrument-specific manner, because in contrast to our finding, a recent study reported a group difference in mechanical detection sensitivity but not in two-point discrimination between mixed classical musicians (string, keyboard, and wind) and non-musicians^29^. A cross-modal coupling of functional adaptation between the somatosensory and auditory systems^30^ further suggests a possibility that the observed superior somatosensory function in the pianists is associated with their superior auditory function, which can be interesting to be addressed in future studies.

In the motor domain, the precision of sustained force production was superior for the pianists compared with the non-musicians (Fig. 1D), which suggests a transfer effect of musical training on fine motor control in non-musical tasks. To the best of our knowledge, this finding is novel. Previous studies have primarily focused on the dynamic aspects of force^31^,^32^ and movement control^28^,^33^,^34^ of the fingers of musicians. A number of neurophysiological studies have provided evidence for structural and functional adaptations of the sensorimotor system, including the motor cortex, somatosensory cortex, and cerebellum, as a result of extensive musical training^1^,^3^. Plastic adaptations of the motor system responsible for force control are likely to play a role in the precise control of non-musical sustained force production in the pianists.

In contrast with the group difference between the pianists and the non-musicians, the inter-individual difference in the fine control of force production during the piano keystrokes among the pianists was accounted for by the individuals’ differences in weight discrimination ability (Fig. 4A). A possible mechanism underlying the correlation of the somatosensory and motor functions is that an innate ability of force discrimination perception determined a learning gain of precise control of force production through musical training, although the present study was not originally designed to address this issue straightfowardly. The evidence supporting this idea includes no group difference in terms of force discrimination ability between the pianists and the non-musicians. There was also no correlation between the somatosensory and motor abilities of the pianists and their histories of musical practice (i.e., early and deliberate musical practice), which might be due to early optimization of these abilities through piano training that was commenced before the critical period (i.e. age 7) in all of the present pianists. In addition, there was no correlation between spatial tactile acuity, in which the effects of musicianship were evident, and fine motor control in the pianists, which does not support an alternative possibility of larger enhancement of motor precision in the pianists who acquired finer somatosensory ability through musical training. Thus, it is presumable that finer force discrimination ability signaled more accurate information about motor error during piano practice and thereby facilitated fine motor control via the feedback error learning^21^. It is also possible that repetitive and prolonged provision of accurate sensory feedback in itself enhanced the ability of precise force control owing to a reciprocal link of neuroplasticity in the somatosensory and motor systems^12^. These postulations are further compatible not only with our observation of no correlations between these somatosensory and motor functions in the non-musicians, who never underwent extensive multimodal musical training, but also with a recent theoretical model of gene-environment interaction^26^.

One may expect that there can be some particular genetic predisposition that commonly determines both the somatosensory and motor functions of pianists, which can explain a correlation of sensory and motor functions across pianists. Several recent studies reported the presence of genetic factors that determine musical expertise^24^,^25^. We also demonstrated that neither the age of starting musical training nor the amount of deliberate practice accounted for the inter-individual differences in the maximum speed of piano performance among skilled pianists^31^. Similarly, neither factors concerning the history of musical practice covaried with both the somatosensory and motor functions in the present pianists. Therefore, it is possible to postulate that pianists with innately superior somatosensory functions are also genetically advantaged in terms of fine motor control. However, this idea is incompatible with our observation of no group difference in weight discrimination ability between the pianists and non-musicians.

An intriguing observation was the correlation between each of the weight and spatial discrimination abilities and precision of sustained force production across the non-musicians (Table 2 and Fig. 3). By contrast, these somatosensory functions were not correlated with the precision of dynamic force production (i.e., increases and decreases in the force level over time). The results suggest that the individuals capable of finer weight and spatial discrimination performed a feedback control of force production in a more elaborate manner. Compared with the trained musicians, the non-musicians relied more on sensory feedback during their finger movements^35^. However, the effects of individual differences in daily hand use cannot be ruled out thoroughly.

The variability in the timing of the constrained piano keystrokes covaried negatively and positively with the loudness variability of the keystrokes in the pianists and the non-musicians, respectively. Because constrained keystrokes require independent control of movements between the fingers, a lack of independent movement control therefore compromises the control of both timing and force^36^. It is therefore likely that the non-musicians with superior independent control exhibited more precise control of timing and force in the constrained piano keystrokes. The opposite finding in the present pianists may suggest that the variability of the timing and force in the task in the experts was not associated with independent control of finger movements. Because the present constrained task was instructed to elicit both timing and loudness as accurately as possible, the results may reflect an individual difference in the attentional focus of these two target variables.

In conclusion, the present study demonstrated that the inter-individual difference in the active force perception ability, which was independent of early and extensive piano training, was associated with that in fine motor control in the pianists. The result suggests that individuals with innately superior sensory function benefited to a greater extent from extensive multimodal musical training and thereby acquired finer motor control. Foster and Zatorre (2010) showed a relationship between structural cortical differences in musicians and the performance on musical transformation tasks, which, however, remained when accounting for the individual amount of musical training^27^. This finding is also in agreement with the idea that training is not the only cause of the observed changes. However, there also remains a possibility that the superior somatosensory-motor functions in the pianists are associated with not quantity but quality of piano practice. Also, a way of calculating the total amount of musical practice may influence the results^37^,^38^,^39^, which should be carefully taken into account in future studies.

## Methods

### Participants

Forty participants took part in the experiments. The participants included twenty-one right-handed pianists (eighteen females and three males) aged between nineteen and thirty-four years (24.3 ± 4.7 years) and nineteen age-matched right-handed non-musicians (fifteen females and four males) aged between 19 and 39 years (21.6 ± 5.2 years). All pianists majored in piano playing and underwent formal musical education at music conservatories, whereas the non-musicians had very little or no experience studying piano playing (less than three years). In accord with the Declaration of Helsinki, the experimental procedures were explained to all participants. Informed consent was obtained from all participants prior to participation in the experiment. The experimental protocol was approved by the ethics committee at Sophia University, and all methods were performed in accordance with the relevant guidelines and regulations. A sample size was determined prior to initiating the whole data collection by means of a power analysis using G-Power.

### Experimental Tasks

The experiments consisted of five sensorimotor function tests (two passive sensory function tests, one active sensory function test, and two motor function tests). Each of the tests was performed with the participant’s non-dominant ring finger to minimize confounding linear additive effects of hand use during daily and recreational activities.

#### Two-Point Discrimination Test

To evaluate spatial discrimination ability, the discrimination threshold of the left ring fingertip was measured via a two-alternative forced-choice simultaneous spatial two-point discrimination task^6^. The participants were asked to place their left hand palm-up on a table and close their eyes. The experimenter gently pressed two sharp points of a measuring compass (Takei Scientific Instruments Co., Tokyo) on each participant’s fingertip. Six pairs of needles with separation distances between 0.5 and 3.0 mm in 0.5-mm steps were used. For a control, zero distance was tested with a single needle. Two series of ascending and descending distances between the points were tested. Each participant answered whether they could perceive the provided stimulus as one point or two points in a binary fashion.

#### Monofilament Test

To evaluate passive force discrimination ability, the cutaneous pressure threshold of the left ring fingertip was measured via a two-alternative forced-choice cutaneous pressure discrimination task using monofilaments (North Coast Medical, Inc.). A total of four Semmes – Weinstein monofilaments (touch–test sensory evaluator; a uniaxial force of 0.008, 0.02, 0.04, and 0.07 g; radius of 1.65–2.83 *μ*s) were used as stimuli. An experimenter pressed a monofilament carefully and gently against the surface of each participant’s left ring fingertip for three seconds. Two series of ascending and descending pressures were tested. Each participant closed his/her eyes and was asked to report whether he/she could perceive contact with the filament.

#### Weight Discriminator Test

To assess active force discrimination ability, the weight sensibility threshold of the left ring fingertip was measured using a set of weights (Takei Scientific Instruments Co., Tokyo). A total of eight different weights (a reference weight of 100 g and comparison weights of +0, +3, +6, +9, +12, +15, +18, and +21 g) were used. The experimenter placed two weights on a table in front of the participant. Each participant was asked to lift up each weight with the fingertip of the left ring finger as slowly as possible, hold for three seconds, and return the weight to the initial position. Each participant then answered whether each of them was heavier or both were the same weight in a two-alternative forced-choice fashion.

#### Music-irrelevant Motor Function Test

We evaluated music-irrelevant motor functions of the left ring finger using a custom-made force sensor system (Leading-Edge Research and Development Accelerator, Inc.). The resolution and maximum measurable force of the sensor were 0.05 and 49N, respectively. A target trajectory of isometric force production was visually displayed on the screen and had a shape of trapezium consisting of three successive phases: increase, hold, and decrease of force. Each of these three phases lasted for eight seconds (i.e., twenty-four seconds in total) and had a peak of 1.6 N. Each participant exerted force on the force sensor with the left ring fingertip in an isometric manner to trace the targeted force trajectory as accurately as possible over five trials. The force data were sampled at 1 kHz. During the isometric force production, the participants were instructed to keep the remaining four digits immobilized.

#### Music-relevant Motor Function Test

Using a digital piano with wooden keys (Yamaha DGP-5), we evaluated music-relevant motor functions of the left ring finger. An experimenter sounded a metronome (loudness: 60 MIDI velocity; tempo: 1 strike per second), and each participant struck a piano key with the left ring fingertip with a *staccato* touch to synchronize with the metronome and elicit the target loudness of tones. Each participant performed the task either by keeping the other four digits immobilized and depressing the adjacent keys (“constrained condition”) or without any explicit requirements regarding the motions of the non-striking fingers (“unconstrained condition”). The participants performed each of the two conditions over five trials.

#### Questionnaire

A short questionnaire that included questions about his/her history of musical training (i.e., age at which musical training was initiated and average practice time at each age) was completed by each participant.

### Data Analysis

To identify a sensory discrimination threshold according to the results of the individual sensory tests, a logistic regression analysis was performed on the derived datasets. The threshold was defined as the effectiveness level computed from a logistic regression.

For the music-irrelevant motor test (i.e., visually guided isometric force production with the fingertip), the absolute value of the difference between the target force trajectory and the force trajectory produced by a participant was computed every millisecond and was then averaged over time for each of the increase, hold, and decrease phases to yield an index for the erroneous force production amount. The computed value was then averaged across five trials.

For the music-relevant motor test (i.e., repetitive piano keystrokes), the absolute value of the difference in each of loudness and timing between the targeted tone provided by the metronome and the tone produced by the participant was computed every second and was then averaged over time for each of the constrained and unconstrained conditions. We used these variables as the indices of timing and force variability of the piano keystrokes.

For each of the individual variables, a two-sample t-test was performed to assess the group difference between the pianists and the non-musicians (p < 0.05). Neither multivariate analysis of variance (MANOVA) nor p-value correction for multiple comparisons was carried out, firstly because each of the individual sensory and motor tests were performed separately and independently, and secondly because we did not hypothesize a difference between any pair of the sensory and motor functions. In addition, a multiple regression analysis was performed to evaluate the covariation between the individual sensory and motor abilities for each of the pianists and non-musicians. The regression model predicted each of the sensory and motor variables based on the remaining variables (p < 0.05). We performed the regression analysis for each of the two groups separately, because we did not have any hypothesis regarding a difference in the regression coefficient between the groups.

## Additional Information

**How to cite this article**: Hosoda, M. and Furuya, S. Shared somatosensory and motor functions in musicians. *Sci. Rep.* **6**, 37632; doi: 10.1038/srep37632 (2016).

**Publisher's note:** Springer Nature remains neutral with regard to jurisdictional claims in published maps and institutional affiliations.

## Figures and Tables

**Table 1:**
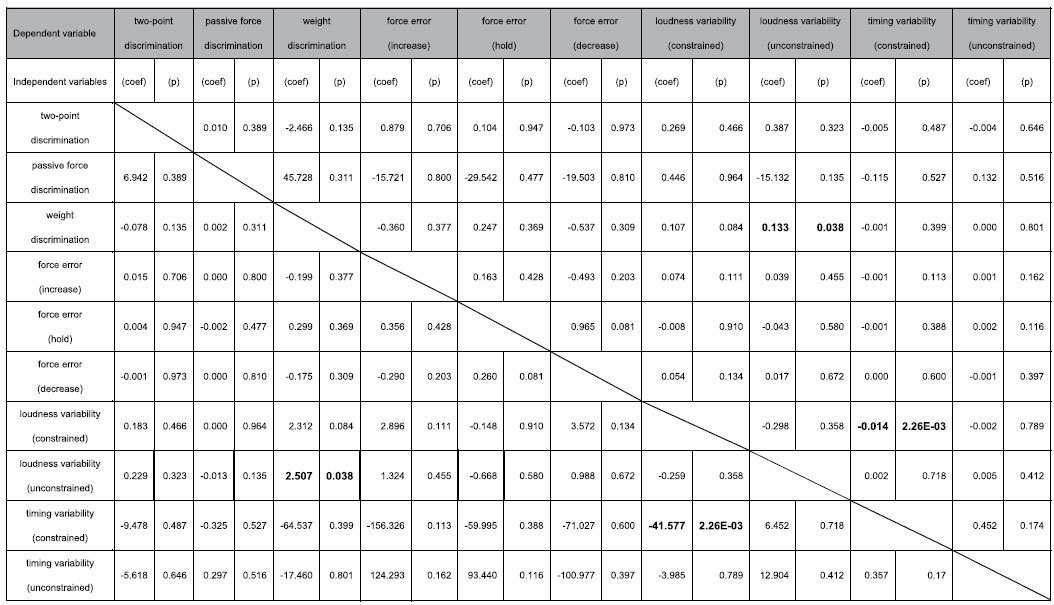
Statistical results of multiple regression analyses between the somatosensory and motor functions in the expert pianists.

(coef) and (p) indicates a partial-regression coefficient and p-value derived from the multiple regression analysis, respectively. Lines indicates independent variables (i.e. predictors), whereas each column indicates each of the dependent variable. A value in bold indicates p < 0.05. E-N indicates × 10^−N^.

**Table 2:**
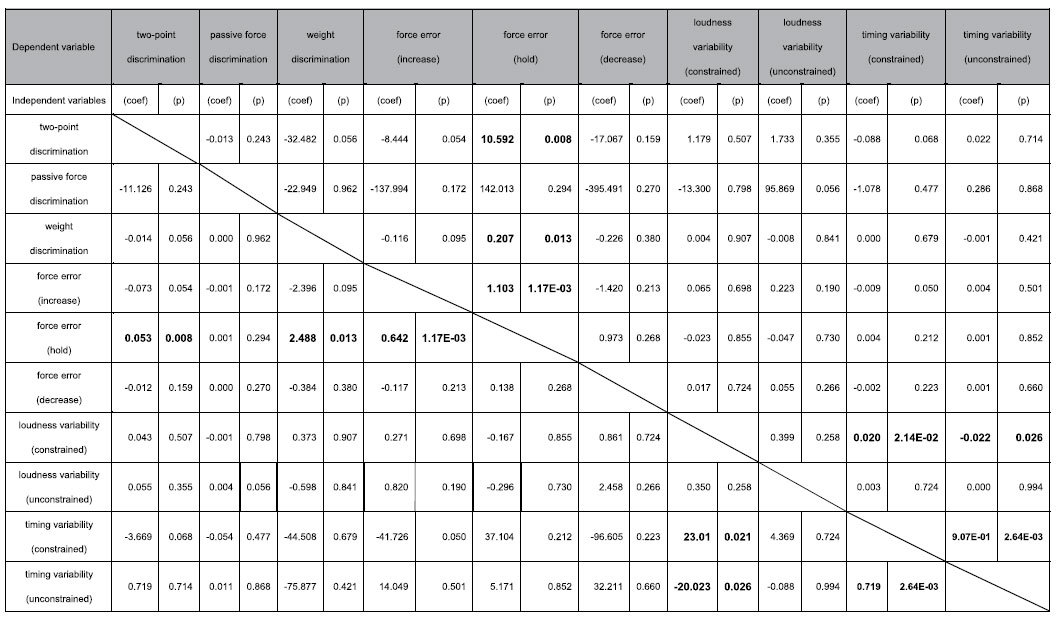
Statistical results of multiple regression analyses between the somatosensory and motor functions in the non-musicians.

(coef) and (p) indicates a partial-regression coefficient and p-value derived from the multiple regression analysis, respectively. Lines indicates independent variables (i.e. predictors), whereas each column indicates each of the dependent variable. A value in bold indicates p < 0.05. E-N indicates × 10^−N^.
